# Laryngo-pharyngeal Tuberculosis

**DOI:** 10.1016/S1808-8694(15)31188-5

**Published:** 2015-10-19

**Authors:** Leonardo Conrado Barbosa de S á, Roberto Campos Meirelles, Ciríaco Cristóvão Tavares Atherino, José Roberto Carvalhaes Fernandes, Fabiana Rocha Ferraz

**Affiliations:** 1 ENT Resident - Pedro Ernesto University Hospital - Ro de Janeiro. M.S Student - General Surgery - Federal University of Rio de Janeiro Medical School - FM-UFRJ; 2 PhD, Adjunct Professor of Otorhinolaryngology - School of Medical Sciences - Universidade do Estado do Rio de Janeiro; 3 PhD, Adjunct Professor of Otorhinolaryngology - School of Medical Sciences - Universidade do Estado do Rio de Janeiro; 4 M.S. Assistant Professor of Otorhinolaryngology - School of Medical Sciences - Universidade do Estado do Rio de Janeiro; 5 Medical Resident - Pedro Ernesto University Hospital - Universidade do Estado do Rio de Janeiro. MD. Otorhinolaryngology Department - Pedro Ernesto University Hospital - da Universidade do Estado do Rio de Janeiro

**Keywords:** extrapulmonary, larynx, granulomatosis, pharynx tuberculosis

## Abstract

Tuberculosis of the Larynx and pharynx only is rare. However, in the last few years, the number of tuberculosis cases in general have had a dramatic increase, thus increasing the possibility of isolated laryngo-pharyngeal lesions.

**Aim:**

To report a case of isolated laryngo-pharyngeal tuberculosis in a pregnant, immunocompetent host. **Case Report**: A 30-year-old pregnant female had complained of odynophagia for the last ten months. There were no other respiratory or systemic symptoms. An oro-pharyngeal granulomatous lesion was found, and the biopsy revealed acid-fast bacilli. There were no clinical or radiologic pulmonary findings. She was submitted successfully to an tuberculosis treatment protocol for nine months.

**Conclusions:**

The authors point out the epidemiological importance of tuberculosis and the need for a higher degree of suspicion when dealing with uncommon upper airway lesions to make an early diagnosis.

## INTRODUCTION

Tuberculosis rarely affects the pharynx or the larynx, especially during pregnancy. Morgagni described the first laryngeal lesions caused by tuberculosis in the XVII century, after carrying out numerous dissections^1^. Later, Lieutaud and Borsieri1 defined the initial concepts of pharyngo-laryngo-pulmonary tisiology, establishing a correlation between the lesions described in the larynx and pharynx with those found in the lungs.

In 1820, Bayle and Broussais1 described an atypical tuberculosis case in the laryngeal mucosa. In the middle of the XIX century, Louis1 established the relations between laryngeal and pulmonary tuberculosis and the basis of its physiopathology, while Rokitansky^1^ carried out the anatomic and pathologic descriptions of the lesions. The larynx mirror, developed by Garcia in 1854 added much from the clinical standpoint to the studies carried out about laryngeal tuberculosis.

In the early XX century, pharyngo-laryngeal tuberculosis, most of the times associated with a pulmonary cavity, became more frequent, representing a sign of disease severity, killing the patient in 70% of the cases^1^.

During the 40's and 50's, after the development of treatments with varied drugs, such as isoniazid and streptomycin, the incidence of pulmonary tuberculosis reduced, and the pharyngo-laryngeal involvement became less frequent.

Nonetheless, in the last two decades, thanks to a drop in the quality of treatment and supervision of tuberculosis cases world wide, AIDS as a global epidemic and the development of multi-resistant strains of the Koch bacillus, we are seeing a progressive increase in the number of cases. This situation increases the possibility of having more pharyngo-laryngeal lesions, either associated or alone.

Kalindoros^2^, presented an incidence of 33% of isolated infection in the larynx; however, much lower percentages have been described by other authors in most of the studies found in the literature^2^,^3^. The primary pharyngo-laryngeal form of the disease is still very rare, with very few cases described in the literature^4^, ^5^, ^6^.

The goal of the present investigation is to describe a case of pharyngo-laryngeal tuberculosis without pulmonary involvement, in a pregnant patient, highlighting the importance of a high degree of suspicion when we have lesions in the upper airways for the early diagnosis of tuberculosis in high prevalence areas for such disease.

## CASE REPORT

V.S., female, 30 years old, came to our outpatient ward complaining of oro-pharyngeal pain for 10 months that worsen when she ate. She did not report weight loss, anorexia, cough, fever, sudoresis, chills, halitosis, rhinorrhea, secretion or sputum, pharyngeal pruritus, dyspnea or hemoptysis. She had not had pulmonary diseases or surgeries in the past. She reported having had contact with a patient with pulmonary tuberculosis two years ago. She did not smoke nor drank alcoholic beverages.

**Figure 1:**
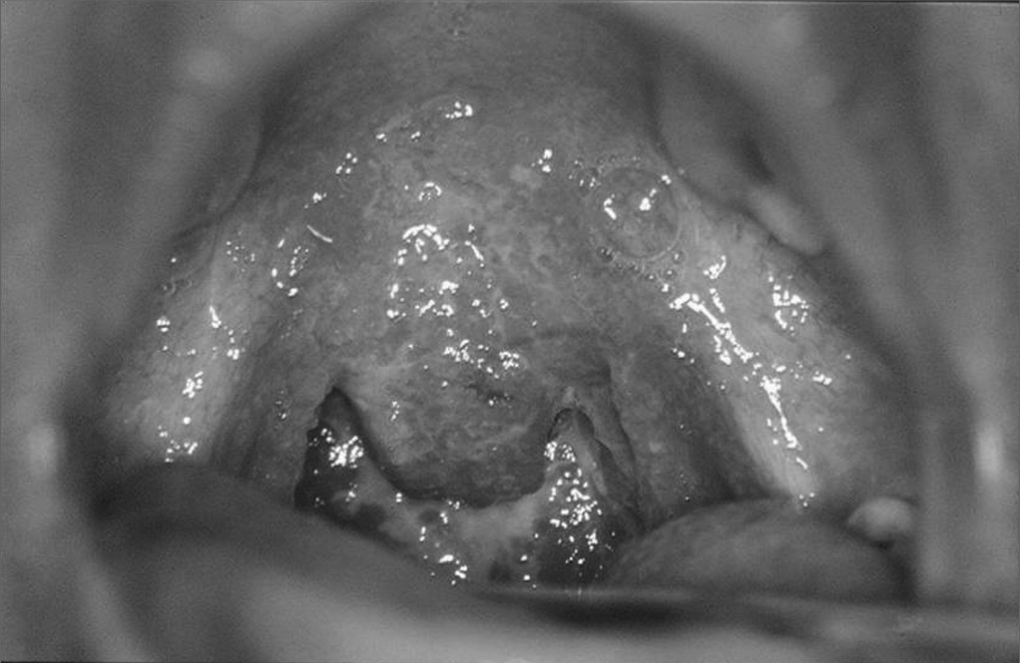
Granulomatous infiltrate in the oropharynx.

Physical exam: 16-week pregnant patient had soft palpable and painless nodes in her left sternocleidomastoid region, without signs of infection or fistula. In the oropharyngoscopy we noticed a granulomatous, hyperemic, friable lesion, involving the soft palate, uvula, tonsils and the posterior pharyngeal wall, especially on the right side, without secretions or ulcerations (Figure 1). The rest of her oral cavity did not show alterations.

Indirect laryngoscopy showed a granulomatous infiltrate in the epiglottis, arytenoids domes, especially to the right and on the right side ventricular fold.

We removed tissue fragments from her soft palate for pathology exam, direct view and culture for fungi, alcohol-acid resistant bacilli and common germs.

Histopathology exam revealed a chronic granulomatous inflammatory process, with alcohol-acid resistant bacilli under Ziehl-Neelsen stain, matching tuberculosis signs. Culture was positive for the Koch's bacillus after 30 days of inoculation.

**Figure 2:**
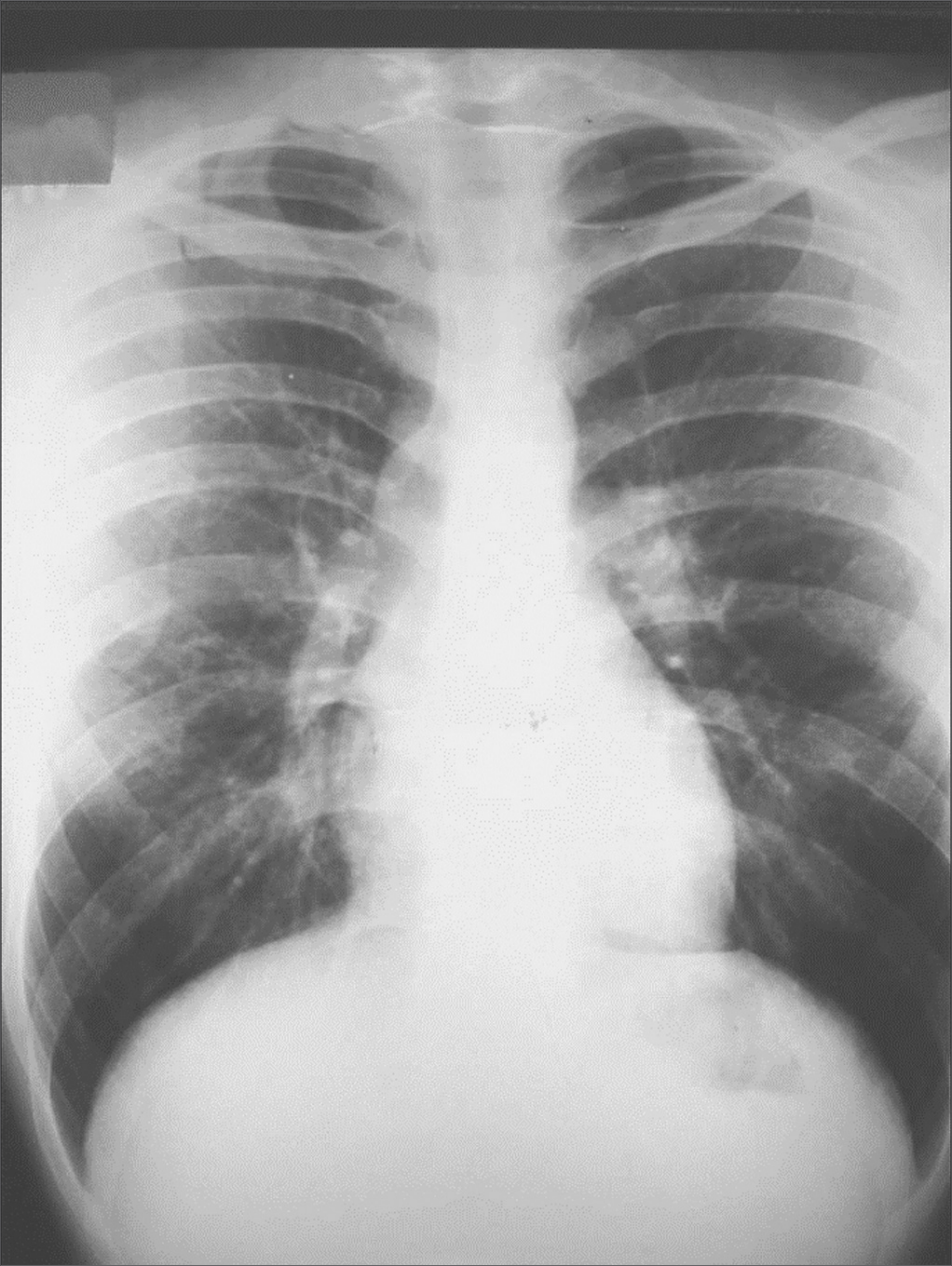
Normal chest x-ray.

Pneumology did not reveal clinical or radiological alterations (Figure 2).

**Figure 3:**
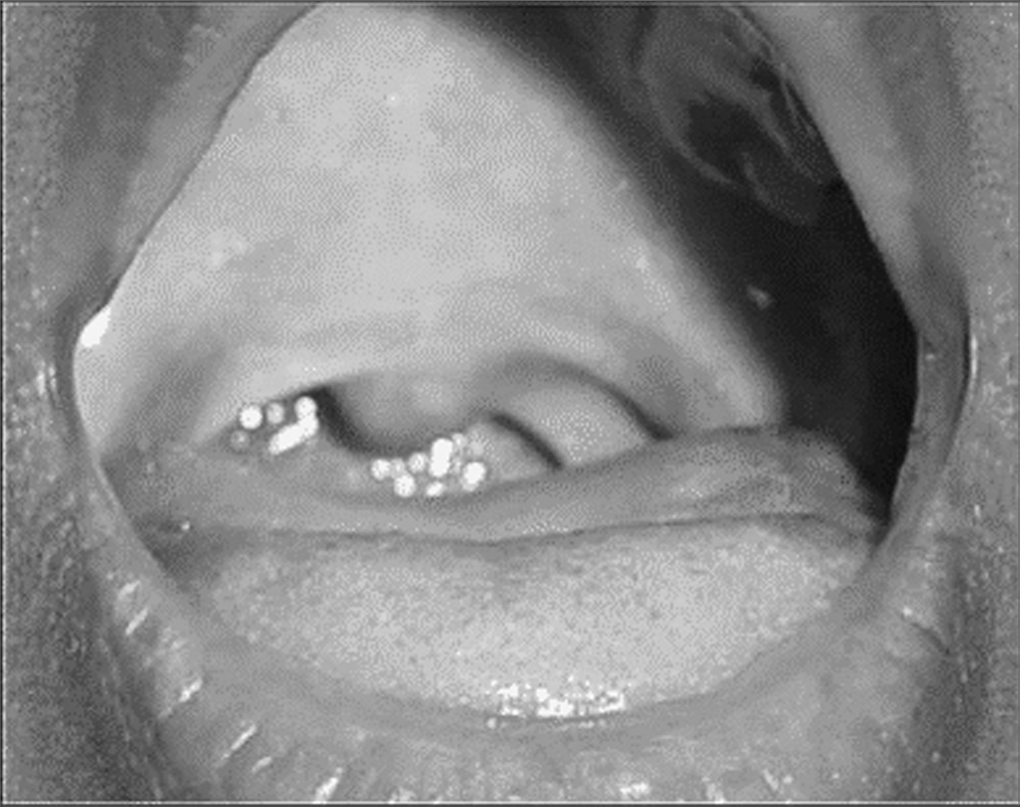
Oropharynx after treatment.

We started with anti-tuberculosis treatment with a triple scheme based on rifampin, isoniazid and pyrazinamide. Her oropharyngeal pain resolved in the first month of treatment. However, after six months, she still had sporadic pharyngeal discomfort and showed soft palate and right arytenoid with hyperemia. We then decided to maintain her treatment for three more months. After nine months of treatment, the patient was asymptomatic and did not show alterations in her clinical exam (Figure 3). She has been followed for one year now, without signs of disease recurrence.

## DISCUSSION

In 1993, the World Health Organization declared tuberculosis a global emergency. In the last decade, there were about 31 million deaths caused by this disease or by its complications, showing a worsening of the situation.

Tuberculosis is an infection caused by the Koch' Bacillus (KB) which affect mainly the lungs and, secondary to that, the genital-urinary tract, hematopoietic organ, central nervous system and upper aero-digestive pathways. The transmission happens when the person contacts bacillary patients who cough the bacillus in Pfluger's droplets, causing pulmonary inoculation or that of another site. At the entry door, usually pulmonary, the KB causes a granulomatous lesion: the inoculation cancrum, which is an epithelioid follicle with giant cells, rich in bacilli. There is also central necrosis evolving to a caseous formation. Later on, the lesion becomes encapsulated. The bacilli may then remain quiescent. Some, captured by the lymphatics, cause a satellite lymphadenopathy, within which the same alterations are produced. This primo-infection at the end of one to two months brings about tuberculin immunization, turning the PPD test positive. Then, the individual becomes refractory to the new infection and to the dissemination of the ongoing infection. This type of immunity is primarily cellular, T-cell mediated and helped by macrophages. From this point, there is definitive cure in 90% of the cases when the bacillary population is small and the host is immunocompetent, with persistence of the pulmonary cancrum and the hilar lymphnode, both calcified. The tuberculin reaction remains positive for the entire life of the individual.

Apparent cure happens in 5% of the cases from the initial disease - cancrum and adenopathy. In these cases, the bacillus may spread through the blood current and induce micro formation of live bacilli that may remain latent for many years. All the organs of the body can be contaminated, especially the well vascularized ones, such as the pulmonary apexes, vertebrae and the renal cortex. From these latent foci and immunodepression, tuberculosis may happen by reactivation.

In most cases, pharyngeal and laryngeal tuberculosis represent an evolution of pulmonary tuberculosis. Numerous hypothesis attempt to explain the infection pathways of these sites, and the blood is the one most accepted. The airborne theory of contamination, proposed by Louis^1^, advocate that the bacilli present in cough would directly contaminate the mucosa, while in the lymphatic hypotheses presented by Kiej^1^, there would be a retrograde contamination, coming from the tracheobronchial lymphnodes, which try to explain the frequent ipsilateral involvement seen in laryngeal and pulmonary lesions.

Laryngeal involvement has been observed in 15 to 37% of the cases of pulmonary tuberculosis, but as primary involvement in only 19% of the tuberculosis cases^7^.

What usually happens is that late reactivation of a laryngeal focus happens during the blood spread phase of the primal infection. During the reactivation process, it is rare to have the involvement of upper airways without pulmonary lesions, exactly because of the complex physiopathology described.

Oropharyngeal involvement has two peaks of incidence, at 30 and at 60 years^8^, the tonsils compartment is the one most affected8, frequently causing dysphagia and odynophagia, often times making up the only complaints of the patient. Morphology varies; there are chronic ulcerated lesions such as painful and bulged tonsils, non-friable, painless uni or bilateral tonsil hypertrophy with hyperemia or necroses. The form described as tuberculosis lymph node comes out as unilateral hypertrophy of the palatine tonsil, pallid in color, followed by subdigastric lymph node disease.

Our patient had diffuse lesion involving not only the palatine tonsils, but also the tonsil pillars, part of the soft palate and uvula, granulomatous and hyperemic. Hypopharynx involvement happens from an extension of the laryngeal tuberculosis.

The laryngeal involvement in the present case, without dysphonia or cough seems strange, because of the very exuberance of the lesions described. In the literature^1^,^7^, ^8^, ^9^, mild progressive dysphonia are reported in 85% of the cases, odynophagia in 45 to 90% of the cases, dry cough, globus pharyngeal and stridor in the more severe cases. Hemoptysis and general body symptoms (weight loss, mild morning fever, sweat and asthenia) are less common. Dyspnea is uncommon in the laryngeal involvement without pulmonary lesion. Odynophagia severity is related to the degree of laryngeal involvement, especially the epiglottis, and is very important for the differential diagnosis of larynx carcinoma, which rarely causes pain.

By decreasing incidence rate, the laryngeal lesions are located on the vocal folds, the ventricular folds, epiglottis, subglottis and posterior commisure^1^, varying from edema and hyperemia similar to a chronic laryngitis all the way to the ulcerated and infiltrating forms, corresponding to the most advanced stage of the disease, with surface epithelium necrosis, ill-outlined borders and foci of caseification. Currently, the infiltrating and pseudotumoral forms are more frequent than the ulcerated ones^3^,^9^, ^10^, ^11^.

The larynx is usually mobile.

Tuberculosis in the upper airway is of difficult diagnosis because of the large variety of lesions aforedescribed. In most cases, the initial diagnosis is of laryngeal carcinoma, because of the clinical presentation and the macroscopic aspect of the lesion. The suspicion of tuberculosis usually stems from a chest x-ray ordered in the preoperative evaluation for laryngeal microsurgery^7^,^12^.

The high prevalence of tuberculosis in Brazil, especially in Rio de Janeiro, led us to biopsy this lesion in the soft palate for direct search of the bacillus by means of the Ziehl-Neelsen stain, and through Löwenstein-Jensen medium culture, which is specific for KB growth. Although it happened in this case, finding the bacilli in direct view is uncommon, because of the low quantity of free bacilli. The culture grows slowly, with average response between two and four weeks. Only after eight weeks, the culture can be considered negative. Identifying the specific type depends on biochemical characteristics.

The routine exam by pneumology and tisiology is fundamental for a complete assessment of the case. For our surprise, the patient did not have any alteration in her clinical exam or in her chest x-ray. Thus, it was not justifiable to proceed with pulmonary investigations.

The treatment used for her is the one recommended by the Brazilian Department of Health with the three drugs: isoniazid (H), Rifampin ® and Pyrazinamide (P), all of them in the first two months and RH for four more months, making up 6 months of treatment. As it happened in this case, treatment may extend to one year. According to the literature, under treatment the patient has a fast clinical improvement with odynophagia remission in the first month. The granulomatous, ulcerated and exudative lesions regress faster than the infiltrative, fibrosis and tumoral ones. Our patient had odynophagia and dysphagia remission, but since she had occasional oropharyngeal discomfort, mild soft palate hyperemia and right arytenoid, we decided to maintain isoniazid and rifampin for three more months, when se enjoyed disease remission.

This case is similar to the diffuse form described in the literature, having association with extensive lesions that affect the glottis and supraglottis, especially posteriorly, hypo and oropharynx, with a granulomatous aspect, mosaic type, and the laryngeal mucosa with extensive hyperemia and covered by muco-purulent secretions.

The major issues pertaining to this case are the pharyngeal involvement - extremely rare, no pulmonary lesions and the fact that the patient was in her fourth month of pregnancy.

Some authors consider pregnancy as a factor associated with the development of tuberculosis,^1^,^8^ especially in countries associated with high malnutrition rates, however no controlled study shows this characteristic as an isolated causal factor.

## CONCLUSIONS

In recent years, AIDS has increased the number of pulmonary and extra-pulmonary cases of tuberculosis. This boom of multi-resistant strains of bacilli to drug therapy has cropped up because of an increase in the incidence, inadequate treatment, treatment non-compliance, thus raising the epidemiological importance of this disease.

Although tuberculosis is a common disease in the Brazilian population, its pharyngo-laryngeal variation is extremely rare, especially when dissociated from the pulmonary form, requiring a high degree of suspicion for diagnosis. Nonetheless, pharyngo-laryngeal involvement in the presence of pulmonary disease seems to be much more common than reported, thus proving the need for further studies.
